# Pilates and Cognitive Stimulation in Dual Task an Intervention Protocol to Improve Functional Abilities and Minimize the Rate of Age-Related Cognitive Decline in Postmenopausal Women

**DOI:** 10.3390/ijerph192013333

**Published:** 2022-10-16

**Authors:** Daniel José Fontel da Silva, Juliana Lima Torres, Luiza Pimentel Ericeira, Naina Yuki Vieira Jardim, Victor Oliveira da Costa, Josilayne Patrícia Ramos Carvalho, Paola Geaninne Reis Corrêa, João Bento-Torres, Cristovam Wanderley Picanço-Diniz, Natáli Valim Oliver Bento-Torres

**Affiliations:** 1 Graduate Program in Human Movement Sciences, Federal University of Pará, Belém 66075-110, Brazil; 2 Neurodegeneration and Infection Research Laboratory, Institute of Biological Science, João de Barros Barreto University Hospital, Federal University of Pará, Belém 66073-005, Brazil

**Keywords:** multitasking behavior, exercise movement technique, cognition, physical functional performance, Pilates, postmenopause, preventive care, rehabilitation techniques

## Abstract

It is already known the effectiveness of Pilates training on cognitive and functional abilities. It is also known that dual-task exercise and cognitive stimuli improve cognition and functional capacity. However, no previous report combined cognitive stimuli and Pilates in dual task and measured its effects on the cognitive and physical performances of postmenopausal women. Objective: To apply an interventional dual-task (PILATES-COG) protocol and to evaluate its influence on memory, language, and functional physical performances on healthy, community-dwelling postmenopausal older women. Methods: 47 women with amenorrhea for at least 12 months participated in this study. Those allocated on the PILATES-COG group underwent a 12-week, twice a week regimen of 50 min sessions of simultaneous mat Pilates exercise program and cognitive tasks. Cognitive and physical functional performance were assessed. Two-way mixed ANOVA was used for data analysis, and Bonferroni post hoc tests were used for within- and between-group comparisons. Results: The PILATES-COG group showed significant improvement after the intervention in semantic verbal fluency (*p* < 0.001; ηρ² = 0.268), phonological verbal fluency (*p* < 0.019; ηρ² = 0.143), immediate memory (*p* < 0.001; ηρ² = 0.258), evocation memory (*p* < 0.001 ηρ² = 0.282), lower-limb muscle strength (*p* < 0.001; ηρ² = 0.447), balance (*p* < 0.001; ηρ² = 0.398), and dual-ask cost (*p* < 0.05; ηρ² = 0.111) assessments on healthy, community-dwelling postmenopausal older women. Conclusion: This is the first report of a feasible and effective approach using Pilates and cognitive stimulation in dual task for the reduction of age-related cognitive decline and the improvement of physical functional performance in healthy postmenopausal women.

## 1. Introduction

Multiple variables contribute to age-related cognitive decline, but significant individual variability is observed [1] as a function of differential influences of nutrition [2], physical activity [1,3], social engagement [4], genetic variability [5], and epigenetic changes [6]. To amplify cognitive performance or mitigate the rate of age-related cognitive decline, social interaction, regular physical activity, and cognitive stimulation have been used [1,4]. There is no consensus, however, about type and duration of exercise programs to improve or mitigate cognitive decline [7,8] and yet less is known about the combined effects of exercise and cognitive stimuli in dual task to improve cognition and functional capacity in healthy [9,10,11,12] or unhealthy [13,14,15] older adults.

It is important to highlight that many activities of daily living (ADL) are performed as dual task and that performing two task simultaneously demands cognitive and motor skills [16] and places higher demands on dynamic balance [17]. The secondary cognitive task seems to interfere on the primary motor task [18]: the dual-task cost (DTC). DTC increases with aging, affecting mobility and cognitive performance [19,20], and dual-task gait performance in older adults is now suggested as a predictive tool for cognitive impairment [21].

The dual task training—combining a motor and a cognitive task simultaneously—seem to be more beneficial for balance, postural control, and cognition and for reducing risk of falls than the practice of exercise performed alone or sequentially [9,22,23,24,25,26]. Different exercise modalities have been investigated on dual-task interventions [9,23,27,28], but there are no studies exploring Pilates as physical exercise and cognitive stimulation in a dual-task paradigm.

Recent analysis on the effects of mat Pilates among older adults showed benefits of its practice for dynamic balance [29,30], lower limb muscle strength [31], hip and lower back flexibility [32], and cardiorespiratory fitness [33].

Due to the safeness, adaptability, and promising exercise approach of Pilates for muscle strength, balance, functional mobility, and postural stability in older adults [31,34,35]; because it shows good acceptance and adherence by older adults, especially female patients [36,37]; and considering that there are no published studies on Pilates in dual task, the main aim of this study was to evaluate the effects of a dual-task intervention based on mat Pilates exercises and multidomain cognitive stimulation on the cognitive and physical-functional performance of postmenopausal older women. Our hypothesis was that a Pilates dual-task intervention would improve memory, balance, lower limb muscle strength, and mobility and reduce the cost of dual-task performance in older women.

## 2. Materials and Methods

### 2.1. Trial Design

This study was a nonrandomized clinical trial designed to assess the effects of an intervention protocol composed of mat Pilates and multisensory cognitive stimulation (PILATES-COG) in dual task on cognitive and physical functional performance of postmenopausal older women. This study was approved by the Institutional Review Board—Hospital Universitario João de Barros Barreto (No. 2146662) and was registered at the Brazilian Registry of Clinical Trials (UTN code: U1111-1237-6670).

### 2.2. Eligibility Criteria

Healthy community-dwelling women who were amenorrheic for 12 or more months were invited to participate by advertisement at churches, social media, and senior centers. The inclusion criteria allowed the participation of women aged 50 years or older, with normal scores on the Mini Mental State Examination (MMSE) adjusted for schooling [38] and a report of a sedentary lifestyle for at least 6 months.

Participants were excluded if they reported previous stroke; balance and/or coordination disorders; physical limitation to exercises practices [32]; or use of medications that may compromise cognitive and functional performance or depression, assessed by Geriatric Depression Scale (GDS-5) [39].

### 2.3. PILATES-COG Protocol and Control Group

Mat Pilates exercises associated with multisensory cognitive stimulation PILATES-COG group were compared with a control group. The PILATES-COG protocol consisted of 24 group sessions, twice a week, 50 min each. Participants were organized in classes of a maximum of 10 participants to ensure appropriated supervision and orientation to each participant.

Each intervention session was conducted by two certified physical therapists with previous experience in the Pilates method. Additionally, an undergrad student supported the sessions as an assistant. Verbal command and individual feedback for the correct and safe motor and cognitive task execution were systematically provided. The exercises chosen for this protocol focused on dynamic balance, hip and lower back flexibility, and trunk and upper and lower limb strength training [32]. Sessions were divided into three phases: warm-up phase with global stretching (5 min); strengthening and flexibility phase with global strength and flexibility exercises (40 min; 2 sets of 8–12 repetitions); and cool-down/relaxation phase with breathing exercises and massage delivered by the therapists with a Bobath ball (5 min) [36,37].

The participants used balls, sticks, circles, and their own body weight as exercise load. Participants were instructed to exercise to their self-limits. Personalized adaptations were made by the physical therapist to ensure the best movement execution according to their own capacities, maintaining the same exercise goal to all participants. Load progressions were adapted to each individual and established through exercise adjustments to increase its difficulty face an adequate performance or improvement on execution. For example, Shoulder Bridge was initially carried out with full lower limb support and progressed for one leg support and later using an unstable surface like the Bobath ball for leg support.

The simultaneous cognitive stimulation protocol was applied during strengthening and flexibility phase, and it was based on previous protocols published elsewhere [9,40]. The cognitive task involved memory, speech, verbal fluency, visual and auditory stimuli, attention, and inhibition. The detailed protocol is shown in Table 1.

The control group (Control) participants received educational materials on health-related topics. Physical activity levels were assessed with the International Physical Activity Questionnaire (IPAQ) [41]. Both groups performed assessment sessions before and after 12 weeks [31,36,37], and they were instructed to maintain their daily routines.

### 2.4. Primary Outcome

The cognitive performance was defined as the primary outcome. Memory and language were assessed with the Word List Memory test from the Battery Consortium to Establish a Registry for Alzheimer’s Disease (CERAD) and Semantic (SVF) and Phonological (PVF) Verbal Fluency test, respectively. These tests were previously used to assess memory and language functions in older adults [42,43,44].

The CERAD word list was used to assess episodic verbal memory (immediate memory and word list delayed recall) and recognition memory. Briefly, for immediate memory assessment, a researcher read aloud a 10-word list, and then participant was asked to evoke as many words as possible, scoring 1 point for each correct one. This procedure was repeated two more times, and the final score was calculated by sum of the three attempts score. The reference cut-off score is 13 points. For the word list delayed recall (evocation memory) assessment, the participant was asked to recall the previous wordlist after a 5-min interval. Each recovered word scored 1 point, for a maximum of 10, with a cut-off score of 3 points. For the recognition memory assessment, a researcher read a list of 20 words, including the 10 words from the initial list and 10 new words. Participant was asked to identify the words from the original list. The cut-off score is 7 points. The criteria to assess CERAD word tests were based on previous norms published elsewhere [45].

Semantic verbal fluency (SVF) was calculated from the average number of words evoked from animal and fruits categories. For each category, participant was asked to mention as many fruits or animal as she could remember for 60 s. The same procedure was made for the Phonological Verbal Fluency (PVF) assessment, for words with the initial sound “A” or “F” as categories. The cut-off points for both fluency tests adjusted to schooling were: <9 points for illiterate, <12 (1–7 years), and <13 (7 years) [43].

### 2.5. Secondary Outcome

Physical functional performance was defined as the secondary outcome. It included lower-limb strength (30-s Chair Stand Test—30 CST), balance (mini Balance Evaluation Systems Test—mini-BESTest), functional mobility (TUG and TUG with dual-task—TUGDT), and Dual-task cost (%) [12,46,47,48,49].

To perform 30 CST, participant was required to seat in an armless chair, with hips, knees, and ankles positioned at a 90º angle and arms crossed in front of the torso. Participant was asked to stand up and sit as many times as possible for 30 s. The number of all completed stand and sit movements was registered. More repetitions indicates better performance (higher lower-limb strength resistance) [49]. Reference mean values for middle aged and older women with age range from 55–64 years is 12.7 repetitions; 65–74 years is 10.7 repetitions; 75–84 years is 9.2 repetitions [50].

The mini-BESTest assesses dynamic balance in a 10 to 15 min examination and contains 14 items divided into four sections: anticipatory postural adjustments; postural responses; sensory orientation; and balance during gait. Each item scores from 0 (worst performance) to 2 points (best performance), resulting in a total score of 28 points. Performance lower than 22 points are indicative of higher fall risk, and higher than 27 low fall risk [51,52].

Functional mobility was assessed with TUG, a practical and useful tool capable of measuring balance and functional performance [48,49]. For TUG assessments, participants were initially positioned as described for 30 CST and instructed to rise from the armless chair, walk 3 m, and return to sit position. A shorter completion time indicates better performance, and the score of 13.5 s is often used as the cut-off point to indicate higher fall risk in older adults [53]. The test was performed twice, and the best performance was used for statistical analysis.

Functional mobility in the dual-task context was assessed with the TUGDT [20,54]. In addition to the commands described for the TUG, participants were asked to speak aloud names of animals while performing the test. Previous data reported elsewhere [20] showed the TUGDT completion time varied from 10.97 and 11.66 s among healthy older adults. Two trials were performed, and the fastest one was used for statistical analysis.

We also estimated the dual-task cost (DTC), which measures the impact from the interaction between motor and cognitive task, currently used as a task abilities indicator [47]. We calculated DTC using formula (1), in which the single- and dual-task times were the time spent on TUG and TUG-DT, respectively; higher score indicates worse performance on the dual task than the single task. DTC ranged from 20 to 30% among middle-aged adults (40–55 years old) and 30 to 40% among older adults (65–85 years old) on TUG combined with serial subtractions of 3 and 7 respectively [20].

DTC = (Dual Task Time − Single Task Time)/(Single Task Time) × 100%(1)

### 2.6. Sample Size

Sample size was calculated using G-Power 3.1 software. The required sample was determined based on data reported elsewhere [36]. Considering their later findings of small-to-moderate effect sizes for cognitive and functional abilities after a Pilates program with postmenopausal women, we estimated an effect size of f = 0.25. Statistical power of 0.90% and a significance level of 95% were applied, resulting in a total sample size estimation of 46 participants.

### 2.7. Allocation

The allocation was made by convenience. Participants had the opportunity to choose to take part in the PILATES-COG group or the control group based on their transport possibilities and personal commitment with the session schedule [9]. Participants received proper explanations about the research aims, procedures, and periods, and they signed written informed consent before initial assessments. First, all the cognitive followed by physical functional performance assessments were completed on the same day, before and after the intervention period.

### 2.8. Statistical Analyses

We carried out statistical analysis using IBM SPSS Statistics software, version 20 (Armonk, NY, USA: IBM Corporation). Student’s t tests for independent groups were calculated to compare age and education between groups. The Shapiro–Wilk test was applied, and extreme outliers were removed if necessary to achieve normality distribution. Levene’s test of equality of variances was applied to verify the homogeneity of variances. After checking these assumptions, we did remove outliers for TUG, TUG-DT, and dual-task cost.

Two-way mixed ANOVA was applied to analyze the main effects of the two independent variables (“Time” and “Group”) and to analyze possible interactions (“Time” x“Group”) on the dependent variable (cognitive and balance performance). In case of interactions, one-way ANOVA was conducted for each independent variable to check simple main effects. In this study, the results from one-way and two-way mixed ANOVA were consistent, so we adopted the latter. Bonferroni post hoc tests were performed to analyze the differences between (PILATES-COG × Control) and within subjects (Pre- × Post-intervention). Effect sizes were calculated as partial eta squared (ηρ²) for within-group effects and described as small (ηρ² = 0.01), medium (ηρ² = 0.06), or large (ηρ² = 0.14) [55].

## 3. Results

**Figure 1:**
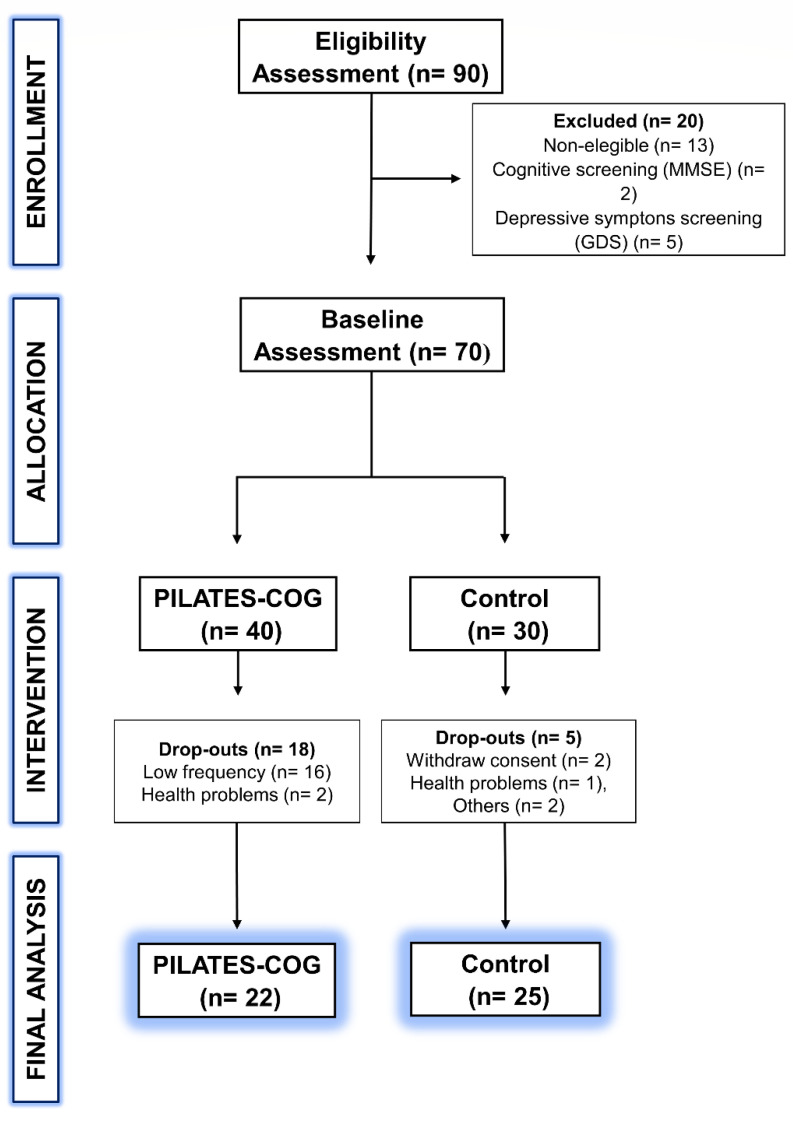
Study Flowchart. Contains information about enrollment, allocation, and inclusion of participants for data analysis.

A total of 47 participants (PILATES-COG: 22 participants; Control: 25 participants) aged between 53 to 83 years old completed assessments and were included in the statistical analysis (Figure 1). Participants of Pilates group had 100% attendance on intervention sessions. Groups were matched by age (PILATES-COG: 66.92 ± 5.49 years of age; Control: 66.09 ± 8.47 years of age) and education (PILATES-COG: 8.88 ± 4.34 years of education; Control: 10.09 ± 3.83 years of education) (Table 2). Both groups were composed mainly by widow (PILATES-COG: 22.7%; Control: 52%) and married women (PILATES-COG: 59.1%; Control: 32%). No adverse effects or complications related to the intervention occurred.

### 3.1. Cognitive Performance Results

**Figure 2:**
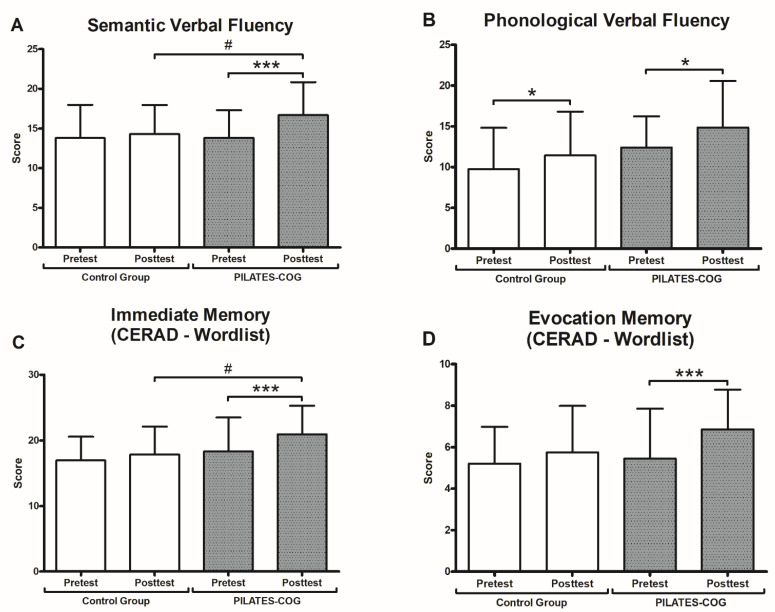
Cognitive Performance. Values presented as mean ± SD. * = *p* < 0.05; *** = *p* < 0.001 (Pre × Post Test). # = *p* < 0.05 (Post × Post Test). CERAD = Consortium to Establish a Registry for Alzheimer’s Disease. (**A**,**B**) Verbal Fluency. (**C**) Immediate Memory. (**D**) Evocation Memory.

The primary outcome results showed a positive influence of Pilates on language (SVF and PFV) and memory (immediate memory and delayed recall) functions (Figure 2).

Main effects of time were found for both semantic (F_(1,37)_ = 11.498, *p* = 0.002, ηp² = 0.237) and phonological fluency (F_(1,36)_ = 10.702, *p* = 0.002, ηp² = 0.229). A group × time interaction was found only for SVF (F_(1,37)_ = 6.256, *p* = 0.017, ηp² = 0.145). PILATES-COG improved performance after the intervention for SFV (*p* = 0.01; ηρ² = 0.268) and PFV (*p* = 0.019; ηρ² = 0.143) while Control showed improvements for PFV (*p* = 0.036; ηρ² = 0.116) but not for SVF (*p* = 0.462; ηρ² = 0.015).

Main effects of time were found for immediate memory (F_(1,45)_ = 14.807, *p* < 0.001, ηp² = 0.248) and evocation memory (F_(1,44)_ = 17.272, *p* = 0.0001, ηp² = 0.282) but not for recognition memory. PILATES-COG improved performance after the intervention for immediate (*p* < 0.001; ηρ² = 0.258) and evocation memory (*p* <0.001; ηρ² = 0.282) but not for the recognition memory (*p* = 0.118; ηρ² = 0.055). The control group did not change over time for any CERAD Battery measures (immediate memory: *p* = 0.166; evocation memory: *p* = 0.102; recognition memory: *p* = 0.545).

### 3.2. Physical Functional Performance Results

**Figure 3:**
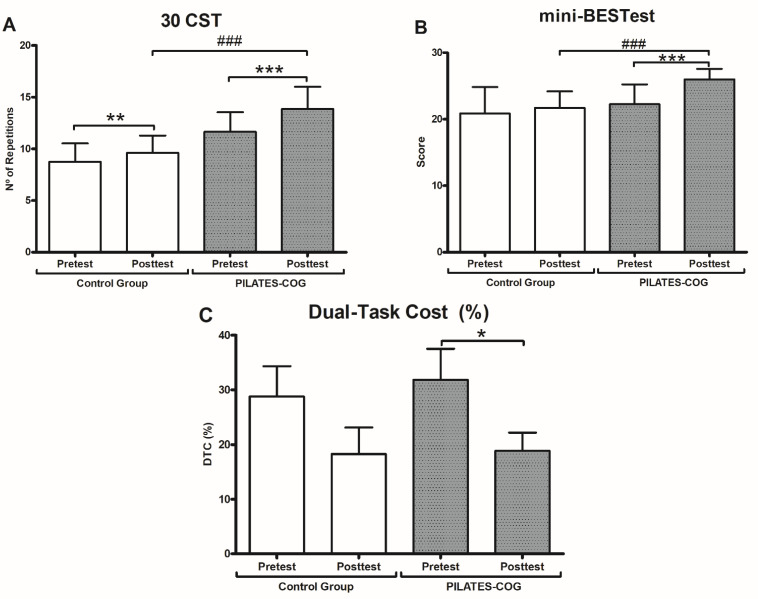
Physical functional performance. Values presented as mean ± SD. * = *p* < 0.05; ** = *p* < 0.01; *** = *p* < 0.001 (Pre × Post Test). ### = *p* < 0.01 (Post × Post Test). 30 CST = 30 s Sit-To-Stand Test. (**A**) Lower-Limb Muscle Strength. (**B**) Dynamic Balance. (**C**) Dual-Task Cost (%).

Two-way mixed ANOVA showed improvements in lower-limb muscle strength, dynamic balance, and reduction of DTC (%) (Figure 3).

For lower-limb muscle strength, main effects of time (F_(1,35)_ = 34.093, *p* < 0.001, ηp² = 0.493), group (F_(1,35)_ = 39.675, *p* < 0.001, ηp² = 0.531), and group x time interaction (F_(1,35)_ = 7.839, *p* = 0.008, ηp² = 0.161) were detected, which means that this variable changed differently at each group over time. Post hoc analyses showed significant increases from baseline in 30 CST performance for both PILATES-COG (*p* < 0.001; ηρ² = 0.447) and Control (*p* = 0.011; ηρ² = 0.170).

For balance assessment with mini-BESTest, results showed main effects of time (F_(1,41)_ = 19.999, *p* < 0.001, ηp² = 0.328), group (F_(1,41)_ = 16.134, *p* = *p* < 0.001, ηp² = 0.282), and the group × time interaction (F_(1,41)_ = 6.482, *p* = 0.015, ηp² = 0.156). Dynamic balance was improved for the PILATES-COG (*p* <0.001; ηρ² = 0.398), and no changes were detected for the control group (*p* = 0.249; ηρ² = 0.032).

Regarding the functional mobility assessments, main effects of group for TUG (F_(1,39)_ = 81.549, *p* < 0.001, ηp² = 0.676) and TUG-DT (F_(1,36)_ = 20.424, *p* < 0.001, ηp² = 0.362) were found, however no pre-post intervention changes were detected for neither PILATES-COG (TUG: *p* = 0.973; TUG-DT: *p* = 0.234) or Control groups (TUG: *p* = 0.731; TUG-DT: *p* = 0.167).

The results for the dual-task costs showed main effects of time (F_(1,36)_ = 6.971, *p* = 0.012, ηp² = 0.162). Post hoc analysis indicated improvements in dual-task ability for PILATES-COG (*p* = 0.041; ηρ² = 0.111) with a reduction of 12.97% on dual-task cost. Control did not have a significant DTC change over time (*p* = 0.112).

## 4. Discussion

The main goal of the present study was to evaluate the effects of 24 sessions of simultaneous mat Pilates and cognitive training on the cognitive function and physical functional performance of postmenopausal women. The main findings suggest there was an improvement on memory, language, lower-limb muscle strength, dynamic balance, and dual-task cost but not on functional mobility.

Studies on dual-task performance assessment were mostly carried out among older adults [9,25,27,54], but dual-task intervention programs in different age groups remain to be investigated. Although the benefits of Pilates on cognitive function were preliminarily demonstrated [36,56,57,58], no studies explored the effects of Pilates and cognitive stimulation in dual task on physical functional parameters and cognitive outcomes.

The maintenance of cognitive functions is an essential aspect for good quality of life and performance in ADL among older adults [59]. Impairments of memory and verbal fluency can serve as markers for screening age-related cognitive declines and may be adequate longitudinal assessment tools for monitoring the effects of clinical interventions [40,60].

Verbal fluency is a cognitive ability with relative stability during aging, especially in women [61], and declines in memory can be observed from midlife, with accelerated deterioration after 60 years; this is associated with lower hippocampal volume, and lower education [62,63] increases the probability of future dementia and neurodegenerative disorders on postmenopausal women [64].

Our findings show that the groups showed different performance in the assessment of semantic verbal fluency over time, with improvements in the PILATES-COG group, with large effect sizes for semantic and phonemic verbal fluency and immediate and evocation memory but not in recognition memory. Improvements on language performance after Pilates in postmenopausal women [36] and language and memory by dual-task interventions among older adults [9,23,25,65] have already been reported; however, other studies did not find effects after DT interventions in the older adults [65,66].

The priority of the motor task over the cognitive task during interventions, and the nature of cognitive training, may partially explains the divergent results above. In our protocol, the execution of both motor and cognitive task was equally encouraged, and a multidomain cognitive stimulation was provided. To our knowledge, this is the first intervention that evaluated the effects of Pilates on memory in a healthy postmenopausal population. However, the effects of Pilates training on declarative memory of healthy populations still need to be investigated.

The protocol performed in this study consisted of several exercises aiming to increase lower limb muscle strength and balance, involving, squats, planks, and accessories (e.g., bobath ball and sticks), with movements that required coordination, body alignment, and trunk activation, which may have contributed to the improvement of these functions among the participants. Our results are converging with other findings, showing an increase in lower limb muscle strength (CSTS 30) and dynamic balance (mini-BESTest), both with large effect sizes, reinforcing the effectiveness of Pilates method [31,32,36,37,67] and multicomponent training with/without dual task [9,66] among older adults and postmenopausal women.

There were no positive impacts from the intervention on functional mobility on single or dual tasking. Findings of physical exercise with additional cognitive stimulation on functional mobility are conflicting, with benefits [9,22] while other studies found no improvements after dual-task training compared to active group [66] or sedentary controls [25]. A previous meta-analysis [32] found moderate evidence for functional mobility improvement after mat Pilates interventions. A possible explanation for the lack of benefits in the PILATES-COG group in functional mobility may be that performance at baseline influences the outcomes of clinical trials [37]. In our study, the functional mobility of the PILATES-COG group, but not the control group, met the cut-off values for the TUG test [46], and it is possible they no longer exhibited room for significant adaptations to improve functional mobility with the proposed training. Another point to be considered is that, although TUG is widely used, for samples of young seniors, the TUG total time score may not be sensitive enough to reveal the first signs of functional decline [68,69]. Furthermore, studies have shown that for middle-aged adults and young seniors, TUG performance remain relatively stable [20,70], with significant declines in TUG performance in older adults >65 years [70].

In our study, a significant 12.98% reduction in the DT cost for the PILATES-COG group was found. In fact, there seems to be a relationship between worse DT-cost and aging throughout the lifespan when comparing older adults (>65 years), middle adults (40 to 55 years), and young adults (20 to 35 years) [20]. Other differences can be found among younger (60–74 years) and older adults (>75 years) in dual-task performance, with associations between TUG and DT cost observed only for the older group. Previous meta-analysis [71] suggested that physical exercise interventions reduced DT cost among older adults. Despite the fact that we did not find detectable changes for the TUG and TUGDT, we can infer that the relationship between these measures improved significantly with a moderate effect size (ηρ² = 0.111), which may indicate improved dual-task performance after PILATES-COG.

There are some limitations in our study. The objective load monitoring is a limitation generally associated with the Pilates method: Even if current load monitoring protocols are under investigation [72], the evidence related to progression of load in randomized clinical trials is scarce [73] and mostly based on the self-perception from the subjects, which may have underestimated the load progression during Pilates sessions. The non-randomized study design, the loss of participants in the experimental group, and the differences between groups at baseline are also limitations to be considered. Moreover, the cognitive component of the DT cost assessment was not measured, and it may be of importance for better understanding the relationship between the simultaneous cognitive-motor task or even if there was any kind of task prioritization [74], having in mind that it is possible to find a reduction on cognitive DT-cost without any changes in motor DT cost [75]. Future investigations that assess dual-task performance by quantifying both the motor and cognitive task are essential for assessing the effects of this entire protocol.

Unexpected results were found in our study, such as FVF and lower limb strength improvement for the control group. The main hypothesis for the language functions stands on practice effects found for neuropsychological batteries on cognitively healthy older populations without any intervention [76]. Familiarity with the test environment, with the task or procedural learning, and with the development of strategies over time are some of the possible causes of this improvement observed from repetition [77]. However, despite the possible effects of practice, the statistical analysis suggested that the intervention did in fact promote cognitive benefits, since interaction effects were also observed for the SFV, indicating that the groups presented different results over time: The posttest indicated that the PILATES-COG improved, while there were no differences for Control. Furthermore, when analyzing the effect sizes for the cognitive tests, it is possible to observe that the magnitude of the change after three months, as measured by the effect size, is small or medium for all the significant cognitive measures of Control, while it is large for the same cognitive measures in the PILATES-COG group.

Despite the significant statistical change for lower limb strength, there was no change in physical activity levels for the control group (IPAQ) after the intervention period, and posttest mean is under the cut-off values for their group age [46].

Dual-task training may be less engaging due to its challenging nature from the cognitive and physical perspective [78], which may explain the great number of drop-outs of the initial 40 participants on the intervention group (Figure 1). PILATES-COG showed benefits to cognitive and physical function after the intervention, supporting the feasibility and reproducibility of this protocol in a cognitively healthy population and establishing a safe and positive environment for its practice [79].

## 5. Conclusions

This is the first report describing the benefits of mat Pilates and cognitive training in dual task on healthy older adult women population where the cognitive training did not interfere on classical Pilates principles.

Language, memory, lower limb muscle resistance, balance, and dual-task cost in postmenopausal women were benefited by the dual-task approach. We suggest that the adoption of this protocol may be an effective strategy for amplifying cognition or mitigating age-related cognitive decline and improving physical functional performance among healthy postmenopausal women.

## Figures and Tables

**Table 1:** Dual-task Intervention program protocol.

Session	Pilates Exercise	Cognitive Training Simultaneous to Exercise
1 & 2	One Leg Circles, One Leg Up and Down, Side Kicks Up and Down, Side Kick Inner Thigh Lift, Knee to Chest, Shoulder Bridge.	A participant started a grocery store shopping list sequence saying: “I went to the grocery and bought an…” (e.g., apple). The closest participant was asked to repeat the previous statement and add a new item to the shopping list. The process was continuous and items cumulative until everyone in the group had contributed.
3 & 4	Spine and Hamstring Stretch (Standing), Squat + shoulder flexion, One Leg Circles with a stick (Standing), Side Kicks (Hip abduction), Shoulder bridge, Shoulder Bridge with gluteal lift, leg circles and leg Up and Down.	The participants had to speak three words, and then make simple arithmetical task, and then recall the three words previously said.
5 & 6	Shoulder bridge with a ball, leg circles, Leg Up and Down, Bird Dog, roll over and dorsal extension	Serial addition and subtraction calculations of 3, 5, and 7.
7 & 8	Shoulder bridge with a ball, hip flexion holding ball, push-ups with knee support, air squat, leg Up and Down with hip extension (airplane), lunge with a stick	The participants were encouraged to speak letters from the alphabet, intercalate, and/or try listing objects from different rooms of a house.
9 &10	Single Leg Stretch, Leg Inclination, Plank, Rolling Back Down, Plank with Leg lift, spine stretch with stick	The group was asked to listen and pay attention to a song and to complete the lyrics after it was paused. Participants were also asked to mention words present in the lyrics.
11 & 12	Double Leg Stretch, Hip Abduction, Knee Flexion and Extension with Ball, Spine Stretch with a stick	Participants were engaged in a collaborative storytelling. A participant initiated a new story, continued by others one by one
13 & 14	Double Leg Stretch with a ball (roll over), Leg Inclination with a ball, Plank, Plank with leg-lift, Lunge with a ball and stick, Spine Stretch with a stick, Bird Dog, Squats with a ball and shoulder flexion.	A participant started a sequence of grocery store shopping list saying: “I went to the grocery and bought an…” (e.g., apple). The closest participant was asked to repeat the previous statement and add a new item to the shopping list. The process was continuous and items cumulative until everyone in the group had contributed.
15 & 16	Bird Dogs, Plank with a ball, Hip Flexion with extended leg with a ball, Lunge with ball, rolling back, one leg circle, double leg circle	Verbal fluency task: the participants were instructed do evoke as many words as they could remember. The categories were fruits, animals, house objects, or personal names.
17 & 18	Shoulder bridge with leg lift, single leg stretch, spine stretch with a stick, Leg Pull Front, Hip Abduction, Knee Flexion and Extension with ball, Swan, and Swimming	Stroop Test—A word list was displayed. The participants had to read the names of the words instead of their colors.
19 & 20	Bird Dog with a ball, roll up, spine stretch with a stick, single leg stretches, Side Plank, Lunge with a Stick	During the exercise, newspaper news was read out loud by the researchers followed by questions to the participants regarding the information.
21 & 22	Plank with arms on a ball, knee extension sitting on a ball, lunge with a stick, bird dog, squat, and shoulder flexion	Based on researcher tips, participants were asked to guess personal names, song, or objects names.
23 & 24	Knee Flexion and Extension with a ball between the calves, leg inclination with a ball, lunges, squat with the back on the wall.	During the exercise, newspaper news was read out loud by the researchers followed by questions to the participants regarding the information. This activity was intercalated with a serial subtraction by multiples of 6, 7 and 8.

**Table 2:** Cognitive, Physical Functioning, and Dual-task Performance Scores in the Pre- and Postintervention Periods.

Index	Group	Pre- Intervention	Post-Intervention	CI 95%	ηρ²	Interaction (F)	Time (F)	Group (F)
Semantic Verbal Fluency (SVF)	PILATES-COG Control	13.78 ± 3.51 13.80 ± 4.18	16.96 ± 4.14 ***^#^ 14.28 ± 3.66	1.428–4.929 1.790–−0.830	0.268 0.015	6.256 *	11.498 **	1.268
Phonological Verbal Fluency (PVF)	PILATES-COG Control	12.39 ± 3.81 9.75 ± 5.04	14.85 ± 5.69 * 11.41 ± 5.36 *	0.429–4.500 0.112–3.221	0.143 0.116	0.399	10.702 **	3.672
Immediate Memory (CERAD–Wordlist)	PILATES-COG Control	18.32 ± 5.16 16.96 ± 3.61	20.95 ± 4.29 ***^#^ 17.84 ± 4.23	1.294–3.979 −0.379–2.139	0.258 0.042	3.694	14.807 ***	3.583
Evocation Memory (CERAD–Wordlist)	PILATES-COG Control	5.45 ± 2.40 5.21 ± 1.76	6.86 ± 1.91 *** 5.75 ± 2.23	0.726–2.092 −0.113–1.196	0.282 0.060	3.415	17.272 ***	1.420
Recognition Memory (CERAD–Wordlist)	PILATES-COG Control	8.41 ± 1.50 8.42 ± 1.61	8.86 ± 1.16 8.58 ± 1.41	−0.120–1.030 −0.384–0.717	0.055 0.008	0.531	2.474	0.132
Lower-limb strength (CSTS 30)	PILATES-COG Control	11.64 ± 1.90 ^+^ 8.74 ± 1.78	13.86 ± 2.14 ***^###^ 9.61 ± 1.67*	1.369–3.060 0.210–1.529	0.447 0.170	6.482 *	34.093 ***	39.675 ***
Dynamic balance (mini-BESTest)	PILATES-COG Control	22.27 ± 2.96 20.86 ± 3.97	26.00 ± 1.57 ***^###^ 21.71 ± 2.47	2.280–5.174 −0.624–2.388	0.398 0.032	7.839 **	19.999 ***	16.134 ***
Functional mobility (TUG)	PILATES-COG Control	8.16 ± 0.99 ^+^ 11.55 ± 1.79	8.15 ± 1.33 11.68 ± 1.62	−0.677–0.654 −0.593–0.839	<0.001 0.006	0.077	0.053	81.549 ***
Functional mobility with dual-task (TUG-DT)	PILATES-COG Control	10.81 ± 2.13 ^+^ 14.80 ± 4.39	9.92 ± 2.30 13.65 ± 3.45	−2.373–0.598 −2.798–0.504	0.039 0.052	0.056	3.452	20.424 ***
Dual Task Cost (%)	PILATES-COG Control	31.84 ± 25.46 28.76 ± 23.64	18.87 ± 14.79 * 18.27 ± 20.44	0.568–25.372 −2.583–23.562	0.111 0.069	0.078	6.971 *	0.118

Notes: Two-Way mixed ANOVA. Values are presented as mean ± SD. CI (95%) = * *p* < 0.05; ** *p* < 0.01; *** *p* < 0.001. (Pre-test × Post-test); ^#^ *p* < 0.05; ^###^ *p* < 0.001. (Post-Test × Post-Test); ^+^ *p* < 0.001 (Pre-test × Pre-test); ηρ² = partial eta squared; CERAD = Consortium to Establish a Registry for Alzheimer’s Disease; 30CST = 30 s Chair-Stand Test; TUG = Timed Up and Go Test; TUG-DT = Timed Up and Go–Dual Task.

## Data Availability

The raw data supporting the conclusions of this article will be made available by the authors upon request.
